# Treatment of Aggressive T Cell Lymphoblastic Lymphoma/leukemia Using Anti-CD5 CAR T Cells

**DOI:** 10.1007/s12015-020-10092-9

**Published:** 2021-01-06

**Authors:** Jia Feng, Haichan Xu, Andrew Cinquina, Zehua Wu, Qi Chen, Ping Zhang, Xingen Wang, Huiming Shan, Lei Xu, Qian Zhang, Lihua Sun, Wenli Zhang, Kevin G. Pinz, Masayuki Wada, Xun Jiang, William M Hanes, Yupo Ma, Hongyu Zhang

**Affiliations:** 1 Department of Hematology, Peking University Shenzhen Hospital, Shenzhen, People’s Republic of China; 2 iCell Gene Therapeutics LLC Research & Development Division Long Island High Technology Incubator, 25 Health Sciences Drive, Stony Brook, NY 11790 USA; 3 Department of Pathology, Peking University Shenzhen Hospital, Shenzhen, People’s Republic of China; 4 Department of Radiology, Peking University Shenzhen Hospital, Shenzhen, People’s Republic of China

**Keywords:** T cell lymphoma, Hematopoietic cells, CD5 CAR and IL15/IL15sushi

## Abstract

While treatment for B-cell malignancies has been revolutionized through the advent of CAR immunotherapy, similar strategies for T-cell malignancies have been limited. Additionally, T-cell leukemias and lymphomas can commonly metastasize to the CNS, where outcomes are poor and treatment options are associated with severe side effects. Consequently, the development of safer and more effective alternatives for targeting malignant T cells that have invaded the CNS remains clinically important. CD5 CAR has previously been shown to effectively target various T-cell cancers in preclinical studies. As IL-15 strengthens the anti-tumor response, we have modified CD5 CAR to secrete an IL-15/IL-15sushi complex. In a Phase I clinical trial, these CD5-IL15/IL15sushi CAR T cells were tested for safety and efficacy in a patient with refractory T-LBL with CNS infiltration. CD5-IL15/IL15sushi CAR T cells were able to rapidly ablate the CNS lymphoblasts within a few weeks, resulting in the remission of the patient’s lymphoma. Despite the presence of CD5 on normal T cells, the patient only experienced a brief, transient T-cell aplasia. These results suggest that CD5-IL15/IL15sushi CAR T cells may be a safe and useful treatment of T-cell malignancies and may be particularly beneficial for patients with CNS involvement.

Graphical Abstract
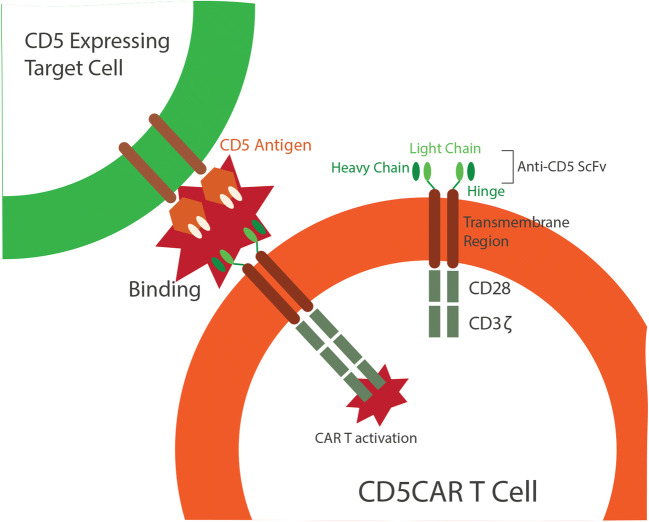

## Introduction

The introduction of chimeric antigen receptor (CAR) immunotherapy has been revolutionary in the treatment of hematological malignancies [1]. While CD19 CARs have had remarkable success in the clinical treatment of B-cell malignancies [2–7], similar strategies for T-cell malignancies have remained largely unexplored. CD5 is a potentially good target for a T-cell based CAR as it is one of the major markers expressed on malignant T cells in T-cell acute lymphoblastic leukemia (T-ALL) and peripheral T-cell lymphoma (PTCL). Additionally, CD5 is not expressed on hematopoietic stem cells and other non-hematopoietic cells, minimizing the risk of off-tumor effects. However, CD5 is also expressed on normal T cells so CD5 CAR T cells may be associated with potential T-cell depletion similar to the depletion of normal B cells seen with CD19 CAR therapy. While B-cell deficiencies can be mitigated through the use of IVIG, there is no such replacement for T-cell deficiencies, causing concern over the higher potential impact of T-cell immunodeficiency. However, irreversible B-cell aplasia is not commonly observed in clinical trials of CD19 CAR [8]. Additionally, preclinical studies have shown that CD5 CAR T cells preferentially target malignant T cells while sparing the normal T population [9]. As CAR T cells are also CD5+, there is also the potential for self-targeting, which would impede therapeutic utility. However, preclinical studies have shown that CD5 CAR T cells downregulate CD5, escaping CAR-mediated self-destruction while still displaying potent lysis of CD5-positive malignant cells [9, 10]. As CD5 is highly-expressed on malignant cells and spared in non-hematopoietic cells, has demonstrated limited T-cell aplasia preclinically, and can escape CAR-mediated self-destruction, CD5 CAR is a potentially useful therapy for T-cell malignancies.

As T-cell malignancies more commonly have CNS involvement than their B-cell counterparts, CNS prophylaxis using high-dose radiation and/or intrathecal chemotherapy is commonly included in the treatment regimen [11–15]. While these prophylactic measures have substantially reduced the burden of CNS-disease, they are associated with significant neurotoxicity and can increase the risk of secondary neoplasms [13, 14]. Even with these preventive treatments, about 5% of patients relapse with isolated CNS disease, representing about 30% of all relapses, and prognostics are poor once CNS recurrence occurs [12, 14]. Due to the limited treatment options available for patients once they relapse with CNS involvement as well as the severe adverse effects of current prophylactic CNS therapy, new clinical strategies to prevent and treat CNS leukemias/lymphomas are an area of clinical interest. CD19 CARs have had success in treating CNS B-cell lymphomas, demonstrating that CAR T-cells can successfully penetrate the blood-brain barrier (BBB) and may be effective therapeutic options for these difficult-to-treat patients [2, 3]. Therefore, a CD5 CAR might also be effective in the treatment of T-cell malignancies that have spread to the CNS or as a prophylactic agent to prevent CNS involvement.

IL-15 is a pleiotropic cytokine that is important for both innate and adaptive immune cell homeostasis. IL-15 is posited to also effect CAR T by increasing T cell numbers through altered metabolic activity and survival, by enhancing effector function (i.e., breaking tolerance to tumor and activating otherwise tolerant T cells), and by promoting the early trafficking of effector and memory T cells to desired locations for therapeutic effect. The IL-15 receptor is composed of an IL-15Rβγ complex that is located on target cells and an IL-15Rα component located on antigen-presenting cells or secreted into the serum [16]. The IL-15Rα can bind to soluble IL-15 with high affinity, and this complex can subsequently bind to IL-15Rβγ to induce its cellular effects [16]. While IL-15 alone has a short half-life, administration of IL-15 linked to soluble IL-15Rα significantly increases its half-life [17], leading to improved *in vivo* tumor response through increased survival of memory T and NK cells [17, 18]. The use of IL-15/IL-15Rα in CAR therapy might augment cancer therapy through enhancing CAR survival, effector function, and effective localization to target tissues. These enhancements could lead to more complete tumor destruction, preventing residual disease or later relapse.

To potentiate CAR through IL-15, we created a CD5 CAR that secretes a soluble IL-15 protein linked to the IL-15Rα sushi domain of the IL-15 receptor (abv. CD5-IL15/IL15sushi CAR). CD5-IL15/IL15sushi CAR T cells demonstrated potent anti-tumor efficacy *in vitro* against a CD5-positive cell line. To test the safety and efficacy of CD5-IL15/IL15sushi CAR, a patient with relapsed T-lymphoblastic lymphoma (T-LBL) with CNS involvement was enrolled in a pilot clinical trial. Remarkably, the administration of CD5-IL15/IL15sushi CAR led to the rapid decline of malignant cells present in the patient’s cerebrospinal fluid (CSF) to undetectable levels within four weeks post-infusion. This led to a rapid resolution of the patient’s debilitating symptoms. Despite the risk of T-cell aplasia, the CD5-IL15/IL15sushi CAR was incredibly specific for malignant cells, and the patient’s CD5 + T cells returned to normal levels around 9 days after CD5-IL15/IL15sushi CAR therapy. The treatment was well-tolerated with no infections reported, and adverse effects were limited to a Grade I CRS. These results indicate that CD5-IL15/IL15sushi CAR may be a useful therapy for T-cell malignancies with CNS involvement, which is more resistant to standard therapies.

## Materials and Methods

### Cell Lines and Materials

Peripheral blood mononuclear cells from healthy donors were obtained from residual samples on a protocol approved by the Institutional Review Board of Stony Brook University. Written, informed consent was obtained from all donors. MOLT4 and HEK293T cell lines were obtained from ATCC (Manassas, VA). T cells were cultured in filtered T cell media, defined as 50% AIM V, 40% RPMI 1640 and 10% FBS, with 1x Pen/Strep (all Gibco, Waltham, MA) and supplemented with IL-2 (300 IU/mL; Peprotech, Rocky Hill, NJ), unless otherwise specified. MOLT4 cell line was cultured in RPMI, 10% FBS, 1x Pen/Strep (Gibco). HEK293T cell line was cultured in DMEM, 10% FBS, 1x Pen/Strep (Gibco), except where noted.

### Lentiviral Vector Production

Lentiviral production was achieved as previously described [19–21]. Briefly, HEK293T cells were cultured in T flasks until 70–80% confluence was reached. Cells were then transfected with the expression plasmid containing CD5-IL15/IL15sushi CAR, and viral packaging plasmids, using the calcium phosphate method (CaCl2 solution, 2xHBS). Cells were incubated with transfection solution in DMEM supplemented with 2% FBS for 6–8 hours, when it was removed and replaced with DMEM with 10% FBS, 50 mM HEPES (Gibco), 1x sodium pyrophosphate (Gibco), sodium butyrate (Millipore, 1 mM), and Pen/Strep. After 24 hours incubation, this supernatant was harvested, and filtered through a 0.2 uM disc filter, and stored short-term at 4^o^C or long-term at -80^o^C.

### Characterization of CAR T Cells

Human PMBC buffy coat cells were activated for 48 hours in the presence of 50 ug/mL anti-human CD3 antibody (Miltenyi) in 50% AIM V, 40% RPMI 1640 culture media supplemented with 10% FBS, Pen/Strep, and 300 IU/mL IL-2. Activated T cells were washed, then transduced with CD5-IL15/IL15sushi CAR lentiviral supernatant. 48 hours after transduction, cells were harvested, washed, and moved to tissue culture plates with fresh media and IL-2, as above. After 2 days incubation, cells were harvested and stained first with goat-anti-mouse F(Ab’)_2_, (Jackson Immunoresearch, West Grove, PA). Cells were then washed and stained with streptavidin-PE conjugate (Jackson) and mouse anti-human CD3, CD5, and CD20 (Tonbo Biosciences, San Diego, CA), washed, suspended in 2% formalin, and analyzed by flow cytometry (FACSCalibur, BD).

### Co-culture Target Cell Ablation Assays

CD5-IL15/IL15susuhi CAR T cells or control T cells (control) were incubated with target cells at ratios of 2:1 (200,000 effector cells to 100,000 target cells) in 1 mL T cell culture media, without IL-2 for 24 h. Target cells were from the MOLT4 cell line. After 24 hours of co-culture, cells were stained with mouse anti-human CD3 and CD5 (Tonbo).

### GMP Manufacturing of CAR T Cells

Manufacturing of CAR T cells was performed in a Good Manufacturing Practice laboratory (GMP-lab). Peripheral blood apheresis PBMCs was obtained from an allogeneic donor (the patient’s sister). PBMCs were then isolated by Ficoll density gradient centrifugation. Pan T cells were activated by incubation with mouse anti-human CD3 antibody (Miltenyi) for 48 hours. Activated T cells were transduced with lentiviral vector CD5-IL15/IL15sushi for 48 hours. After lentiviral transduction, CAR cells were cultured in AIM V/RPMI medium, containing human serum, and 300 IU/mL IL-2 until day 10. Before infusion, the CAR T cells were subjected to detection of pathogenic microorganisms and contaminants (for example, bacteria, fungi, virus, mycoplasma, and endotoxin) to ensure safety. The CAR T cells were also labeled first with goat anti-mouse F(Ab’)2 antibody, then with streptavidin-PE and anti-human CD3-PerCp, and analyzed by flow cytometry, to determine transduction efficiency.

### Patient Information

Written, informed consent for publication was provided by the patient. The patient was first diagnosed with T-LBL in April 2011. He responded to chemotherapy of BFM regimen and subsequently received an allogeneic hematopoietic stem cell transplant (HSCT) donated from his sister. After a progress-free time of 7 years, he relapsed with unilateral testicular enlargement in July 2018. Flow cytometry confirmed T-LBL, and NGS showed TET2, ASXL1, TET1, and CSF3R mutations. No abnormality was found within the bone marrow. After orchiectomy, he was treated with chidamide, a histone deacetylase inhibitor (HDACi), and high-dose chemotherapy (methotrexate, pegaspargase, 6-mercaptopurine). During the follow-up, bone marrow and CSF continued to be negative. In May 2019, a right tibial mass was detected and treated with three-dimensional conformal radiotherapy (3DCRT) with a dose of 3Gy*10F and a cumulative radiotherapy of 6 Gy/2F/2D. Bone marrow MRD was negative.

In August 2019, the patient felt blurred vision, swelling pain, and protrusion in the left eye. CSF analysis revealed scattered lymphoid cells in August 2019, that had progressed to large number of immature cells detected by January 2020. Cerebrospinal MRD in January 2020 revealed 90.71% of abnormal residual leukemia cells. The immunophenotype was CD5+, CD3+, CD99+, CD1ACD56-, CD4-, CD8-, and CD45+. Cerebrospinal fluid examination revealed protein qualitative weakly positive and leukocytes at 270/µL. Cerebrospinal fluid biochemistry demonstrated glucose at 2.7 mmol/L and total protein of 2.49 g/L. PET-CT in January 2020 revealed the inner rectus muscle, inferior rectus muscle, and optic nerve of the left eye were thickened with increased metabolism, which was considered a lymphoma invasion (Deauville score 5 points) 4. The sixth cervical nerve on the right was thickened with increased metabolism, and lymphoma was considered to invade the peripheral nerve (neurolymphomatosis). There was no sign of malignant tumor in the brain. In February 2020, MRI of head and orbit show abnormal signal of left internal carotid artery beside the cavernous sinus and abducens nerve. Enlargement of the medial rectus, superior oblique, inferior rectus, and option nerve in the left eye was considered. Bone marrow aspirate in February 2020 revealed no abnormal cells. Lumbar puncture and sheath injection revealed CSF pressure of 210 mm H_2_O, total cell count of 337 × 10^6^/L, and white blood cell count of 298 × 10^6^/L. Urine/CSF total protein (U/CSP-TP) was 2.47 g/L. Immature cells were found in CSF and a total of 23,000 nucleated cells were analyzed by flow cytometry. CD34+, CD2+, CD5+, CD7+, CD3-, CD56- abnormal T blasts accounted for 96.26% of lymphocytes (95.88% of nuclear cells), and the positive rate of CD38 was 99.18%. In April 2020, the patient was given decitabine (10 mg d1-10) and cedabamine (10 mg, 5 days a week). In March 2020, the patient irreversibly lost vision in his left eye.

After failing to respond to chemotherapy, intrathecal therapy, and HDACi, the patient was enrolled in a clinical trial of CD5-IL15/IL15sushi CAR T cells. The patient showed no GVHD and the chimerism of the T lymphocyte donor was 100%. Before CAR T cell infusion, the patients received two days of treatment with fludarabine (30 mg/m2/day) and cyclophosphamide (300 mg/m2/day) for lymphocyte depletion, which was intended to reduce the tumor burden and T cells, and facilitate engraftment and homeostatic expansion of CAR T cells. The source of the T cells was from the same donor of allogeneic hematopoietic stem cells with HLA matched (the patient’s sister). The patient received a total dose of 2.0 × 10^6^/kg CAR T cells (6.3 × 10^7^ CAR T cells/m2) with split dose in 2 days. Adverse events after CAR T cell infusion were graded according to National Institutes of Health criteria (Common terminology Criteria for Adverse Events, version 4).

## Results

### Characterization of CD5-IL15/IL15sushi CAR

**Fig. 1:**
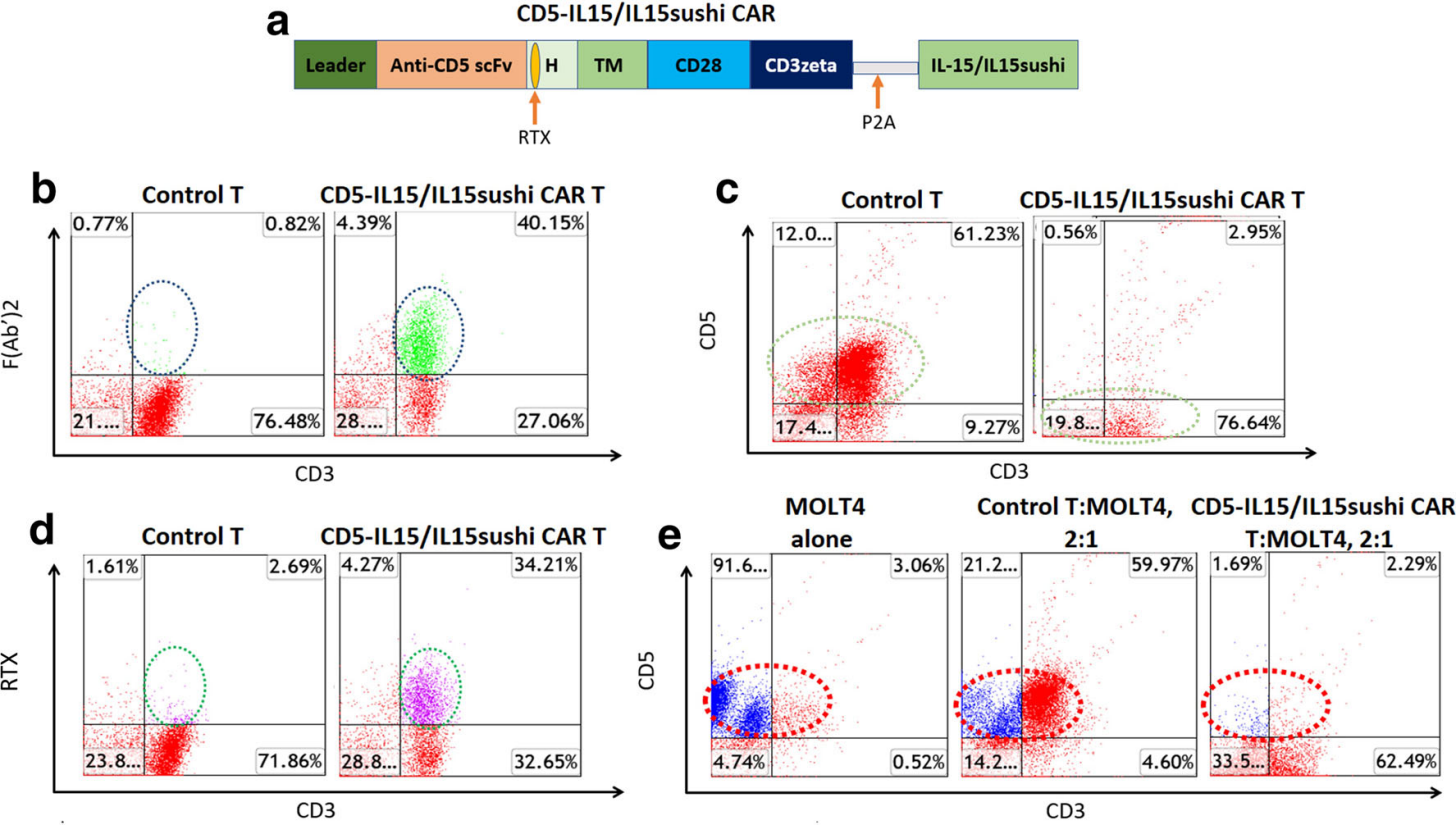
Validation of CD5-IL15/IL15sushi CAR construct. **a** Schematic representation of recombinant lentiviral vector encoding CD5 CAR linked with the P2A self-cleaving sequence to the IL-15/IL-15sushi domain of the IL-15 alpha receptor. Expression is driven by the spleen focus-forming virus (SFFV) promoter. The IL-15/IL-15sushi portion is composed of IL-2 signal peptide fused to IL-15 and linked to sushi domain via a 26-amino acid poly-proline linker. **b** Activated T cells from human peripheral blood buffy coat were transduced with either control (left) or CD5-IL15/IL15sushi CAR (right) viral supernatant from transfected HEK-293FT cells. 48 hours after transduction, cells were harvested, washed, and moved to tissue culture plates with fresh media and IL-2. After 2 days incubation, cells were harvested and stained with goat-anti-mouse F(Ab’), mouse anti-human CD3 and CD5, and against the RTX-binding epitope and analyzed by flow cytometry). Staining with F(Ab’) showed ~40% of T cells expressed CAR following transduction. T cells are displayed in red and transduced population is circled and colored green. **c** Transduced cells demonstrate normal CD3 expression and downregulation of CD5. T cells are colored red and circled. **d** Transduced cells show expression of the rituximab-binding epitopes on the cell surface. T cells are displayed in red and transduced population is circled and colored purple. **e** A co-culture experiment using CD5-positive MOLT4 cells was performed at E:T ratio of 2:1 for 24 hours. Cells were analyzed by flow cytometry using mouse anti-human CD5 and CD3 labeling. During 24-hour co-culture experiments, CD5IL-15/IL-15sushi CAR T cells showed profound killing (92%) of MOLT cells compared to control T cells. MOLT4 cells are blue, T cells are red, and target population is circled

We have previously shown that CD5 CAR demonstrates cytotoxic effects against T cell malignancies *in vitro* and *in vivo* [10, 22]. This CD5 CAR bears a humanized anti-CD5 scFv domain linked to the IL-15/IL-15sushi domain by a P2A self-cleaving sequence. The IL-15/IL-15sushi domain consists of an IL-2 signal peptide fused to IL-15, which is linked to the soluble, sushi domain of the IL-15 α receptor via a 26-amino acid poly-proline linker (Fig. 1a). Two rituximab (RTX)-binding epitope sites are present within the hinge region as a safety switch whereby RTX can be administered to quickly deplete CAR T cells (Fig. 1a). CD5-IL15/IL15sushi CAR was subsequently transduced into T cells. Staining with goat anti-mouse F(Ab’) confirmed surface expression of the CAR and determined a transduction efficiency of 40% (Fig. 1b). Staining against CD3 and CD5 revealed that while transduced T cells retained CD3 expression, CD5-IL15/IL15sushi CAR T cells lost CD5 expression (Fig. 1c), consistent with previous data demonstrating CD5 downregulation, which prevents CAR-mediated self-lysis. Staining against the RTX-binding epitope illustrated a similar transduction efficiency (34%) and confirmed that this region is exposed and capable of binding (Fig. 1d). Finally, to assay the ability of the CD5-IL15/IL15sushi CAR T cells to target CD5 + cells, 24 h co-cultures with either control or CD5-IL15/IL15sushi CAR T cells versus the CD5-expressing MOLT4 line were performed in an effector:target (E:T) ratio of 2:1. Compared to control, CD5-IL15/IL15sushi CAR T cells exhibited almost complete lysis (92%) of MOLT4 cells (Fig. 1e), confirming cytotoxic potential of this CAR against CD5.

### CD5-IL15/IL15sushi CAR T Cells Significantly Reduce Orbital Edema

**Fig. 2:**
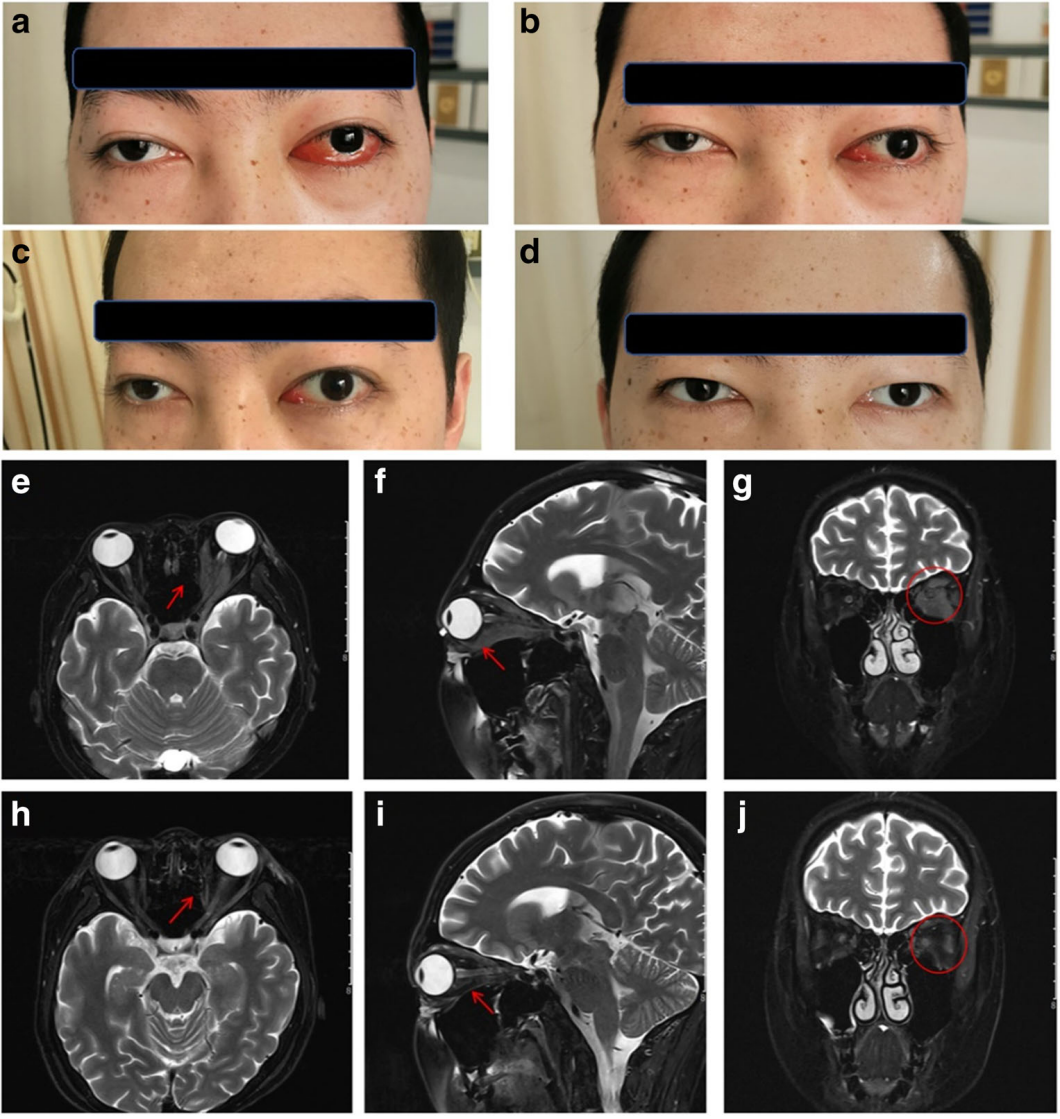
CD5-IL15/IL15sushi CAR T cells rapidly reduce patient’s orbital edema.** a** Prior to CD5-IL15/IL15sushi CAR treatment, the patient’s left eye had significant exophthalmos. Repeat imaging **b** 1 week after CD5-IL15/IL15sushi CAR infusion, **c** 2 weeks after infusion, **d** and 3 weeks following therapy demonstrate rapid resolution of the patient’s swelling. Prior to CD5-IL15/IL15sushi CAR treatment, MRI showed significant edema in the **e** axial, **f** sagittal, **g** and coronal planes. Red arrows and red circle mark the dense soft tissue infiltrate. 8 weeks following CD5-IL15/IL15sushi CAR treatment, repeat MRI imaging shows reduction in the soft tissue mass shadow in the **h** axial, **i** sagittal, **j** and coronal planes

A 22-year-old male with relapsed T-LBL with CNS involvement was recruited for this clinical trial (NCT04594135). After CNS relapse and failure to respond to traditional treatment, the patient was enrolled. The source of the T cells was from the same allogeneic-HSCT donor he received nine years ago (the patient’s sister). Prior to CD5-IL15/IL15sushi CAR treatment, the patient’s left eye showed significant exophthalmos, redness, and swelling (Fig. 2a). Only one week of treatment resulted in the substantial improvement of these signs (Fig. 2b), which continued to improve over the next two weeks (Fig. 2c and d). Three weeks post-infusion, the signs of orbital involvement had completely recovered (Fig. 2d), displaying the incredible ability of CD5-IL15/IL15sushi CAR T cells to ameliorate lymphoma-mediated CSF swelling. Similarly, while MRI prior to treatment demonstrated involvement of the left external eye muscles and optic nerve (Fig. 2e-g), CD5-IL15/IL15sushi CAR therapy led to significant reduction in the soft tissue mass shadow visualized 8 weeks after therapy (Fig. 2h-j). Additionally, before treatment, the patient’s edema caused a severe, debilitating headache. Remarkably, the patient’s headache was relieved within three days after the initiation of CD5-IL15/IL15sushi CAR T cell therapy, displaying the CAR’s ability to cause rapid and profound improvements in the symptoms of CNS lymphoma.

### CD5-IL15/IL15sushi CAR T Cells Drastically Reduce CNS Lymphoblasts

**Fig. 3:**
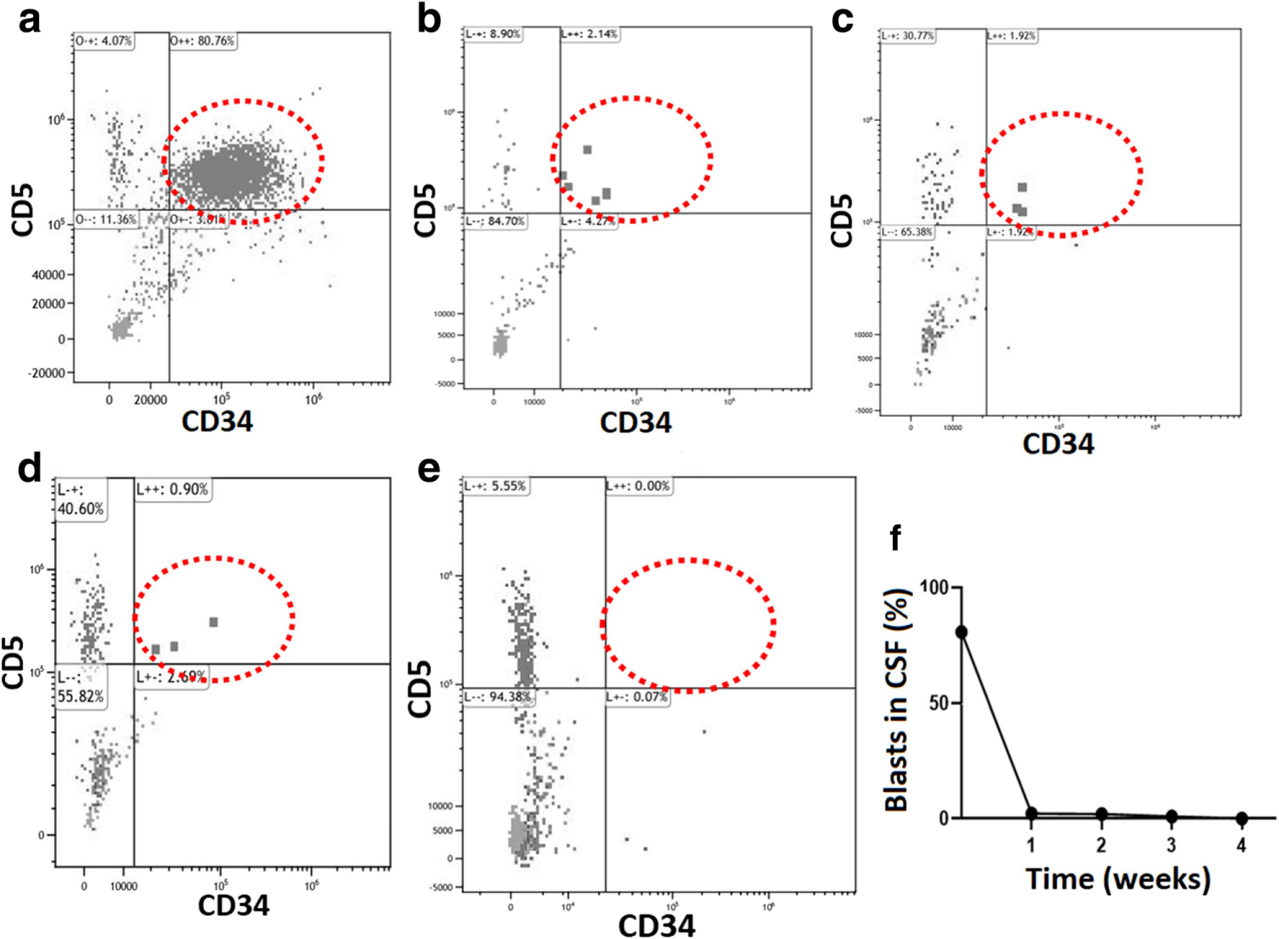
CD5-IL15/IL15sushi CAR T cells display potent lysis of malignant CD5 + lymphoblasts in CSF.** a** Prior to CD5-IL15/IL15sushi CAR T cell therapy, CSF analysis demonstrated a substantial increase in lymphoblasts, accounting for around 80% of the CSF cells, that were almost 100% CD5 + C34+ (circled population). **b** One week following CAR infusion, the lymphoblasts decreased dramatically, now accounting for only about 2% of the patient’s CSF cells. **c** Two weeks following therapy, the percentage had decreased to just under 2%, **d** three weeks after therapy it had decreased to just under 1%, **e** and by four weeks post-CAR, lymphoblasts were undetectable in the CSF. **f** Graphical summary of the CSF lymphoblast population in the weeks after CAR therapy

Next, we analyzed the effect of CD5-IL15/IL15sushi CAR T cells on the malignant lymphoblast population within the patient’s CSF. Flow cytometry analysis of the patient’s CSF prior to CD5-IL15/IL15sushi CAR therapy revealed nearly 80% of the patient’s CSF cells were lymphoblastic cells, and these cells were almost 100% CD5 + CD34+ (Fig. 3a). One week alone of CD5-IL15/IL15sushi CAR T cells led to an extraordinary effect on the lymphoblastic cells, which were reduced to just ~ 2% of the patient’s CSF (Fig. 3b). The lymphoblastic cells continued to decrease over the next few weeks, reaching undetectable levels by four weeks post-CAR infusion (Fig. 3c-e). Figure 3f displays the trend of lymphoblasts throughout the first four weeks of CAR therapy, demonstrating the incredible lytic capability of CD5-IL15/IL15sushi CAR T cells leading to an almost immediate, complete eradication of the patient’s disease in a difficult-to-treat location, the CNS, where current therapies currently have limited effectiveness and severe toxicities.

### CD5-IL15/IL15sushi CAR T Cell Reduces CSF Abnormalities With Limited Systemic Effects

**Fig. 4:**
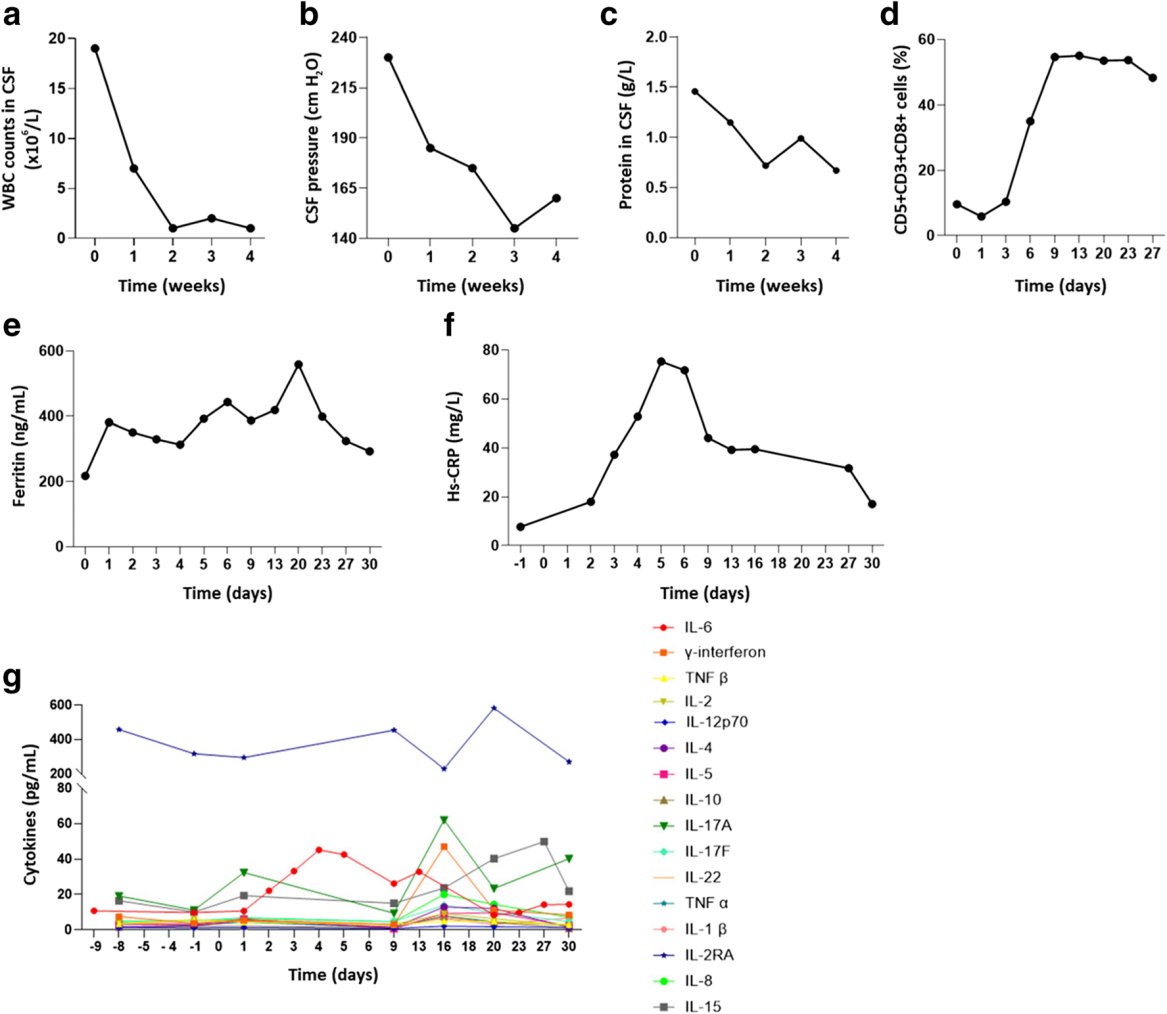
Evaluation of patient’s CSF and serum following CD5-IL15/IL15sushi CAR T cells.** a** CD5-IL15/IL15sushi CAR T cells were able to rapidly reduce the WBC count of the CSF to normal levels (< 5 × 10^6^ cells/L) 2 weeks post-infusion. The reduction of WBCs was associated with marked improvements in the patient’s (**b**) CSF pressure and (**c**) protein levels within the CNS, leading to resolution of the patient’s severe headache. **d** CD5 + CD3 + CD8 + T cells were measured to determine CD5-IL15/IL15sushi CAR T cell’s effect on the patient’s normal T population. As CD5-IL15/IL15sushi CAR T cells are CD5-, the CAR T cell expansion would not affect this measurement. Despite being CD5+, there was remarkably limited T cell aplasia with the T cells returning to normal levels 9 days after CAR injection. Measurement of (**e**) ferritin and (**f**) Hs-CRP, markers of inflammation, demonstrated a mild Grade I CRS toxicity following CAR therapy. Levels returned to normal within 1 month. **g** Measurement of cytokine levels showed remarkably stable cytokine levels following CD5-IL15/IL15sushi CAR T cell therapy, demonstrating a limited and localized response. Importantly, despite secretion of IL15/IL15sushi complex from the CAR T cells, IL-15 levels remained low post-therapy

Prior to CD5-IL15/IL15sushi CAR T cell administration, the WBC count in the patient’s CSF was very elevated (Fig. 4a). Similar to the decline of lymphoblasts following therapy (Fig. 3), the WBC count rapidly declined following treatment, returning to normal levels 2 weeks post-therapy (Fig. 4a). These reductions were associated with declines in the CSF pressure (Fig. 4b) and CSF protein levels (Fig. 4c), correlating with the improvements in the patient’s orbital swelling and headache. As the CD5-IL15/IL15sushi CAR T cells are CD5- (Fig. 1d), analysis of the CD5 + CD34- T population would allow us to isolate the patient’s normal CD5 + T cells, which should be vulnerable to CD5-IL15/IL15sushi CAR T cells. Previous data with our CD5 CAR showed extensive lysis of both malignant and normal CD5 T cells *in vitro* [10], causing concern for the potential of T-cell aplasia in the patient. Incredibly, the CD5 + T cells were only suppressed for the first few days after treatment, rapidly returning to normal levels by Day 9 (Fig. 4d). While CD5-IL15/IL15sushi CAR T cells demonstrated potent lysis of malignant CD5 + lymphoblastic cells (Fig. 3), the normal T cells were remarkably spared. This greatly reduces the risk of prolonged T-cell aplasia and associated infections, suggesting that CD5-IL15/IL15sushi CAR T cells may be a safe way to quickly lead to disease remission. Indeed, there were no infections reported following transfusion, indicating that the transient immunodeficiency might be well-tolerated.

The infusion with CD5-IL15/IL15sushi CAR T cells was well-tolerated by the patient, only eliciting a Grade I CRS toxicity with transient elevations in ferritin (Fig. 4e) and Hs-CRP (Fig. 4f) levels. Similarly, cytokine levels measured during the first month of therapy indicated relatively stable expression (Fig. 4g). Importantly, IL-15 levels did not elevate substantially post-infusion, staying within the pg/mL range (Fig. 4g). As CD5-IL15/IL15sushi CAR T cells secrete a IL15/IL15sushi complex, expansion of the CAR population might lead to excessive levels of systemic IL-15. However, this was not observed in the patient. As excessive levels of IL-15 have been shown to initiate uncontrolled lymphocytic proliferation [23], the low levels measured may mitigate this risk.

## Discussion

Despite the positive results achieved in the treatment of B-cell malignancies with CD19 CARs, studies of CARs directed against T-cell leukemias and lymphomas have been more limited, and patients with relapsed T-ALL display poor outcomes. In a large study of adults with T-ALL, 123 patients (37%) relapsed, and only eight of these patients survived a median time of 5.2 years [24]. Additionally, only 27 patients received allogeneic transplants while 6 received autograft transplants and 90 patients received no transplants [24]. The high proportion of patients receiving no transplants highlights the difficulty in initiating another remission in patients with relapsed disease. Consequently, new strategies are needed so more of these patients can proceed to potentially curative allografts. CD5-IL15/IL15sushi CAR T cell therapy resulted in the rapid and efficient remission of a patient’s relapsed isolated T-LBL CNS disease as assessed by flow cytometry and imaging (Figs. 2 and 3). Due to the remission induced by CD5-IL15/IL15sushi CAR T cells, this patient is currently undergoing a bone marrow transplant (BMT). CD5-IL15/IL15sushi CAR might therefore be useful as a conditioning agent to a curative BMT for patients who previously would not have qualified due to resistant or relapsing disease that is no longer responding to traditional chemotherapy. Alternatively, due to the high toxicities and morbidities associated with BMT, CD5-IL15/IL15sushi CAR T cell therapy may be useful as a standalone treatment option, although future clinical studies are required to determine the effectiveness of the CAR T cells without subsequent BMT.

Therapy is even more difficult for patients with CNS relapse, as the current treatment options are associated with high toxicities and often ultimately fail. Consequently, leukemia/lymphoma with CNS infiltration is associated with a poor prognosis. In a large study of 609 adults with relapsed ALL, the estimated five-year survival was zero for both those who relapsed with CNS disease alone and those who relapsed with both CNS and bone marrow involvement [12]. Due to these poor outcomes, there is a dire need for new therapies for patients with CNS recurrence. Here, the infusion of CD5-IL15/IL15sushi CAR T cell therapy in a patient with T-LBL relapse with CNS involvement led to the remarkable decline of lymphoblastic cells in the CSF from ~ 80% to ~ 2% in just one week, reaching undetectable levels by week four (Fig. 3). These changes were associated with significant improvements in the swelling associated with the patient’s disease (Fig. 2), improving the patient’s orbital edema and severe headache within three weeks and three days, respectively. These results suggest that CD5-IL15/IL15sushi CAR T cell therapy may be a useful therapy for patients with T-cell CNS relapse who are unresponsive to standard treatments or who cannot tolerate the severe side effects associated with them.

Limitations to the development of CAR T-cells directed against T-cell malignancies have included the potential of self-targeting of CAR T cells and T-cell immunodeficiency. As the antigens used to target T-cell malignancies are often shared antigens present on normal T cells, any CAR T cell directed against these antigens may result in self-lysis, limiting the expansion necessary for a clinical effect. Strategies to circumvent this problem have included the disruption of antigen expression in CAR T cells using the CRISPR/Cas9 system and antigen internalization using ER/Golgi retention sequences [25, 26]. However, these approaches may cause off-target effects and involve additional stress and manipulation of CAR T cells, which may limit CAR functionality. On the other hand, CAR T cells directed against CD5 have been shown to downregulate expression of CD5 themselves to retain their survival and proliferative capacities [9, 10], which was found in this study as well (Fig. 1d). In previous studies, only the CD5 CAR-transduced cells displayed this downregulation, and nearby normal T cells did not downregulate CD5 in response to CD5 CAR [10]. The emergence of a CD5- lymphoblastic cell lineage was not observed in this patient, corroborating that the CD5 downregulation mechanism is likely restricted to the CAR cells and may not provide a mechanism of antigen escape, although longer observation would be needed to determine the risk of relapse. Additionally, as IL-15 is a potent stimulator of T cells and NK cells, the presence of a secreted IL15/IL15sushi complex might stimulate the normal host response against the tumor, preventing residual disease or relapse with antigen-negative cells, but this idea requires more testing as well.

Additionally, CAR treatments for T-cell malignancies may be more concerning due a potentially higher impact of T-cell immunodeficiency compared to B-cell immunodeficiency. While CAR CD19-mediated B-cell aplasia might be ameliorated with immunoglobulin therapy, no such alternative exists for T-cell aplasia. As CD5 is expressed on normal T cells, CD5-IL15/IL15sushi CAR T cells could lead to prolonged depletion of T cells. Importantly, while our CD5 CAR had previously demonstrated potent lysis of normal T cells *in vitro* [10], CD5-IL15/IL15sushi CAR T cell infusion in this patient only resulted in transient T-cell aplasia that recovered to normal levels within only nine days (Fig. 4d). Despite the potent destruction of malignant CD5 + cells (Fig. 3), the CD5-IL15/IL15sushi CAR T cells remarkably spared the patient’s normal T cells. The cause of this discrepancy between our *in vitro* results and the rapid recovery of the patient’s normal T cells is unknown. However, this preliminary data show that CD5-IL15/IL15sushi CAR T cell may be a safe therapeutic agent that causes minimal immunodeficiency-related complications. In situations where there is substantial T-cell immunodeficiency or when there is profound cytotoxicity associated with CAR, mechanisms to quickly ablate the CAR T cells may become preferable. Additionally, as IL-15 increases the persistence of memory T cells and can lead to uncontrolled lymphocytic growth [23], the inclusion of IL15/IL15sushi may lead to prolonged aplasia and excessive lymphocytic expansion. However, the measured serum levels of IL-15 in this patient were minimally elevated and remained within the pg/mL range (Fig. 4g), reducing this risk. Similarly, a Phase I and II clinical trial utilizing NK cells transduced with both CD19 CAR and IL-15 demonstrated increased NK persistence with no measurable increase in IL-15 levels over pretreatment values [27]. While systemic injections of IL-15 or IL-15/IL-15Rα, which lead to elevated serum levels, may put patients at risk for associated toxicities, CAR cells may be utilized as a vehicle to deliver the cytokine directly to the tumor microenvironment where they can exert their beneficial effect while minimizing the systemic effects.

While the risk of prolonged T-cell aplasia or IL-15-induced toxicity appears to be limited, safety switches may be incorporated as a precautionary measure. We have previously demonstrated that low-doses of CAMPATH (alemtuzumab), which binds to CD52 and induces cell death, has resulted in rapid and efficient depletion of CAR T cells *in vivo* [10, 28]. This may allow us to quickly ablate CD5-IL15/IL15sushi CAR T cells in situations of excessive lymphocytic growth or prolonged T-cell aplasia, allowing for the regeneration of new CD5 + cells from hematopoietic stem cells to ensure therapeutic safety. Alternatively, CD5-IL15/IL15sushi CAR includes two rituximab (RTX)-binding epitopes in the hinge region. RTX has been routinely used in the treatment of lymphomas, and administration of RTX has previously led to the lysis of other CAR T cells containing RTX-binding epitopes [29]. While both of these methods have proved to be effective in the depletion of CAR T cells *in vivo*, further clinical trials are needed to determine if CAMPATH and/or rituximab is an effective way to restore CD5 generation in patients following symptomatic T-cell aplasia.

While systemic CAMPATH and/or rituximab might allow regeneration of CD5 T cells due to lysis of CD5-IL15/IL15sushi CAR T cells in circulation, it would be unable to penetrate the BBB and eliminate CD5-IL15/IL15sushi CAR T cells present in the CNS. Due to the high occurrence of CNS disease in T-cell malignancies, it may be beneficial to have residual, long-term CD5-IL15/IL15sushi CAR T cells remain in the CNS. Therefore, the use of CAMPATH and/or rituximab might offer a way of allowing the regeneration of CD5 from hematopoietic stem cells while sparing CNS CD5-IL15sushi CAR T cells, which can monitor and prevent CNS disease relapse. As IL-15 increases the persistence of memory T cells, the secretion of IL15/IL15sushi in the CNS may potentiate this effect and lead to longer persistence. However, locally elevated levels of IL-15 in the CSF may, on the other hand, increase uncontrolled lymphocytic proliferation. In this situation, it might be beneficial to undergo intrathecal CAMPATH and/or rituximab to eliminate the CD5-IL15/IL15sushi CAR T cells. More studies, however, are needed to determine whether prolonged CNS CD5-IL15/IL15sushi CAR T cells could be beneficial as a mechanism to prevent relapse or would instead be detrimental due to increased risk of uncontrolled growth. If the need to eradicate the CNS CD5-IL15/IL15sushi CAR T cells emerges, intrathecal CAMPATH and/or rituximab may be safe in limited doses [30, 31], although the efficiency of these antibodies in depleting CAR T cells in the CNS is unknown.

In conclusion, a novel trial of CD5-IL15/IL15sushi CAR T cell in a patient with relapsed T-LBL with CNS involvement demonstrated rapid depletion of patient’s lymphoblastic cells within the CSF. This led to the improvement of the patient’s symptoms and remission of his aggressive disease. While having a potent effect on the malignant lymphoblastic cells, the normal CD5 + T cells remained mostly unaffected, demonstrating that CD5-IL15/IL15sushi CAR T cells could be a fast and safe method of treating patients with difficult-to-treat T cell lymphoblastic lymphoma/leukemia, even involving the CNS.
